# Hybrid male sterility and genome-wide misexpression of male reproductive proteases

**DOI:** 10.1038/srep11976

**Published:** 2015-07-06

**Authors:** Suzanne Gomes, Alberto Civetta

**Affiliations:** 1 Department of Biology, University of Winnipeg 515 Portage Ave, Winnipeg, MB, Canada R3B 2E9

## Abstract

Hybrid male sterility is a common barrier to gene flow between species. Previous studies have posited a link between misregulation of spermatogenesis genes in interspecies hybrids and sterility. However, in the absence of fully fertile control hybrids, it is impossible to differentiate between misregulation associated with sterility *vs*. fast male gene regulatory evolution. Here, we differentiate between these two possibilities using a *D. pseudoobscura* species pair that experiences unidirectional hybrid sterility. We identify genes uniquely misexpressed in sterile hybrid male reproductive tracts via RNA-seq. The sterile male hybrids had more misregulated and more over or under expressed genes relative to parental species than the fertile male hybrids. Proteases were the only gene ontology class overrepresented among uniquely misexpressed genes, with four located within a previously identified hybrid male sterility locus. This result highlights the potential role of a previously unexplored class of genes in interspecific hybrid male sterility and speciation.

Improving our understanding of the process of speciation is a central problem in biology. Speciation requires that reproductive barriers arise to impede free gene flow among nascent species. Among sexually reproducing organisms, interspecies hybrid male sterility is a commonly observed postzygotic isolation barrier. Species which are separated by partial reproductive barriers, such as those that produce only sterile male hybrids, are ideal candidates for identifying changes associated with speciation. Evolutionary geneticists have used a myriad of approaches to establish associations between speciation phenotypes and gene variants^1^. During the last ten years, a series of studies have used genome-wide (microarrays) or gene-specific (qRT-PCR) approaches to compare levels of gene expression in sterile hybrids to fertile parental species. These studies have reported significant misexpression, particularly under expression, in sterile *Drosophila* hybrids relative to parental species for genes of spermatogenesis^2^,^3^,^4^,^5^,^6^. In particular, microarray studies have found an overrepresentation of genes that act on the final steps of sperm individualization and maturation (i.e. spermiogenesis)^2^,^4^. Such observations, given that during spermatogenesis the majority of transcripts accumulate premeiotically, have lent support for the sterility hypothesis which suggests that down regulation of postmeiotic spermatogenesis genes in sterile hybrids is a causative factor of hybrid male sterility^3^.

The use of species pairs in which male hybrids are sterile regardless of the direction of the cross cannot distinguish whether misregulation of gene expression is a condition linked to sterility or a byproduct of incompatibilities between divergent regulatory elements brought together in a hybrid genome (i.e. fast male regulatory divergence). A way around this problem has been the use of backcrosses to generate both fertile and sterile partial hybrids. Studies using this approach have found support for misregulation linked to sterility^3^,^7^ as well as fast male regulatory divergence^8^,^9^. Another approach is to use species pairs that produce unidirectional sterility to compare gene expression between F1 hybrids that are sterile or fertile. Using this method, we have recently shown support for both the sterility and the fast male hypotheses^10^. However, a surprising result was that out of the 13 spermatogenesis genes targeted, none displayed misexpression in hybrids of the *D. p. pseudoobscura* (*D. p. pseudoobscura*) and *D. p. bogotana* (*D. p. bogotana*) cross.

Here we use RNA sequencing in an effort to detect genome-wide differences in regulation associated with sterility in hybrids between *D. p. pseudoobscura* and *D. p. bogotana*, where only hybrids produced by *D. p. bogotana* females are sterile (i.e. unidirectional sterility). The two species are geographically separated, with *D. p. pseudoobscura* found across North America and *D. p. bogotana* restricted to Colombia in South America. The species have diverged about 0.2–0.25 Myr ago, with estimates of genome-wide intergenic sequence divergence per 500 kb ranging approximately between 0.002 and 0.02^11^,^12^.

We found a larger proportion of misregulated genes associated with the sterile than the fertile hybrid condition. Most genes uniquely misregulated in the sterile hybrids were under or over expressed (i.e. transgressive) relative to expression in the parental species, with similar proportions of both over and under expressed genes. In contrast, a larger proportion of genes in the fertile hybrid were expressed additively. An analysis of allele-specific expression differences between species revealed that divergence in regulation is dominated by *cis*-only and *cis*-*trans* effects driving allelic expression in the same direction. Allelic expression in both hybrids was overall positively correlated, but a subset of genes displayed significant changes in expression ratios between hybrids. These genes showed within-chromosome distribution bias, and we highlight a potentially interesting region within the third chromosome. Finally, we did not find significant differences in the proportion of sperm/spermatogenesis genes uniquely misregulated in sterile and fertile hybrids, but we observed an overrepresentation of putative proteases. Interestingly, three such peptidases (GA21772, GA24794, GA24796) and one peptidase inhibitor (GA15722) were located within a previously mapped hybrid male sterility QTL^13^.

## Results

### Differential gene expression between fertile and sterile conditions

We generated over 400 million individual reads from all samples combined. The raw data has been deposited at the Sequences Read Archive (SRA; GenBank) under accession number SRA205377. Slightly more than 310 million reads were mapped to the available *D. p. pseudoobscura* reference genome (Table 1). This has the risk of mapping bias against *D. p. bogotana* derived reads, due to sequence divergence between the species. Three lines of evidence showed that this is unlikely. Firstly, *D. p. bogotana* had a similar percentage of total RNA sequence reads mapped to the reference genome (72.8% for *D. p. pseudoobscura vs*. 74.0% *D. p. bogotana*) (Table 1). Secondly, if the sequence reads were preferentially mapped to the *D. p. pseudoobscura* reference genome, there would be an overrepresentation of genes with higher expression in *D. p. pseudoobscura* than in *D. p. bogotana*, which was not observed (6,839 and 7,075 gene IDs with higher expression in either *D. p. pseudoobscura* or *D. p. bogotana,* respectively) and this pattern held when we only included genes that were significantly differentially expressed between the two parental species (1,014 *vs*. 1,025) (Supplementary Table S1). Finally, the SNP analysis also shows no bias towards one parental species with more gene IDs with higher expression (5,296 *vs.* 4,808) (binomial test, *P* = 1.0) (Supplementary Table S2).

**Figure 1:**
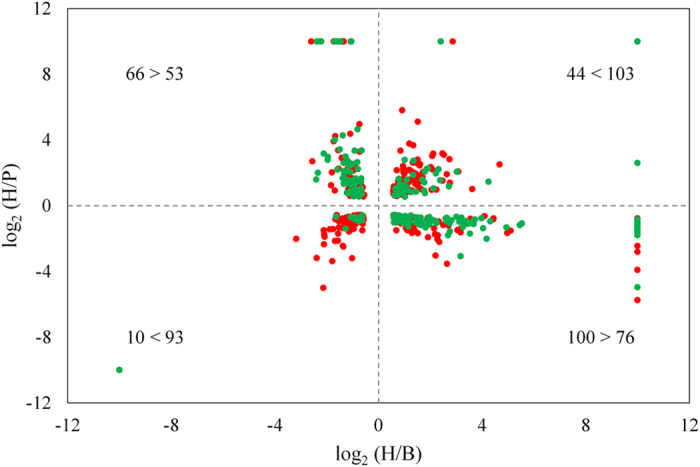
Genes with significant differences in expression between parental species and hybrids. H = hybrids; P = *D. p. pseudoobscura*; B = *D. p. bogotana*. Fertile and sterile hybrid data are represented with green and red circles respectively. Log_2_ ratio values are capped at 10. The number of genes is shown in each quadrant and partitioned into fertile and sterile hybrids respectively. The upper right and lower left quadrants show significantly over and under expressed genes (transgressive) with the two other quadrants indicating additive effects.

Differential gene expression assays were conducted on gene IDs with a minimum of 10 mapped reads in at least one sample (13,928 gene IDs, Supplementary Table S1). Seventy seven percent of gene IDs showed no pairwise differences in gene expression (Supplementary Table S1). Of the remaining 23% of genes showing at least one significant pairwise difference, 2,039 (14.6%) were differentially expressed between the two parental species. Comparison of expression levels between hybrids and parental species showed significantly more gene misregulation in sterile (325) than fertile hybrids (220) (Z = 6.36; P < 0.001). The sterile hybrids also showed a higher proportion of transgressive misregulation (over and under expression) (Z = 12.7; P < 0.001) and lower proportion of additive expression (Z = 3.1; P = 0.002) than the fertile hybrids relative to parental species (Fig. 1). In the context of hybrid male sterility, misregulation unique to each hybrid is informative for the purpose of identifying potential sterility-linked functional groups, pathways, or modes of misregulation. There were significantly more gene IDs uniquely misexpressed in the sterile hybrid (164) than in the fertile hybrid (64) (Z = 9.37; P < 0.001). The fertile hybrid had a significantly larger proportion of additive genes (34) than the sterile hybrid (17) (Z = 3.37; P < 0.001) while the sterile hybrid showed significantly more genes (147) with uniquely transgressive expression than the fertile hybrids (30) (Z = 12.44; P < 0.001) (Supplementary Table S3a).

**Figure 2:**
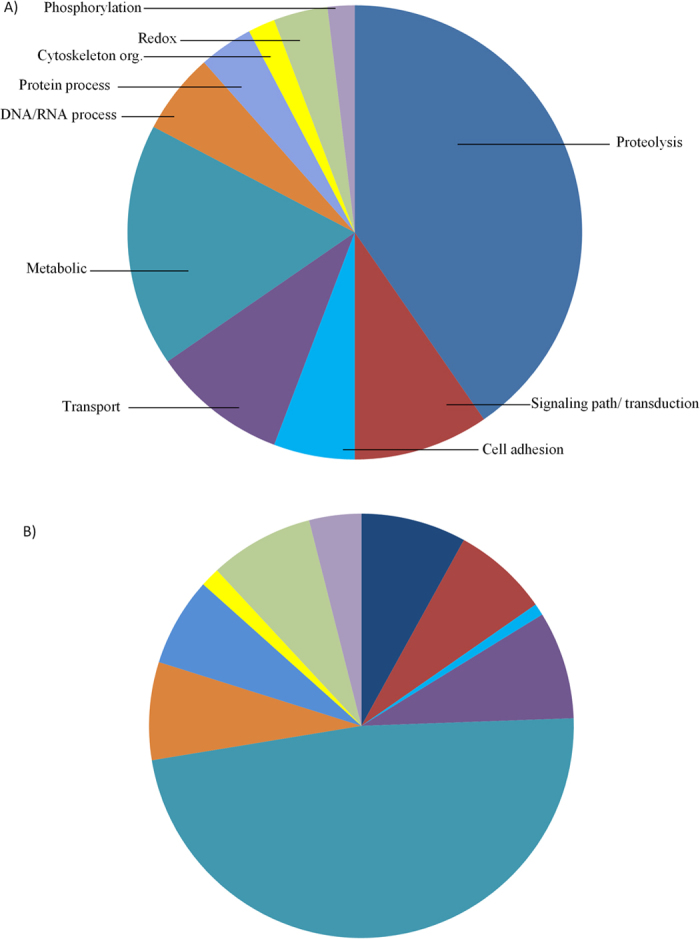
Proportion of uniquely misregulated genes per biological process in the sterile hybrid (A) and the genome-wide expected proportions of each biological process (B). Both proteases and cell adhesive genes were proportionally enriched.

We identified inferred biological and molecular functions of genes uniquely misregulated in sterile hybrids. There was an overrepresentation of the biological process ‘proteolysis’ (13 out of 44 genes) (Fig. 2) as well as the molecular function ‘peptidase/ peptidase inhibitor’ (18 out of 57) relative to other classes (Supplementary Table S3b). The large number of proteases among misregulated genes might be a condition linked to the hybrid nature of the genome. However, fertile hybrids had only 1 out of 30 (3.3%) misregulated genes with an identifiable peptidase domain compared to 20 out of 87 (23.0%) in the sterile hybrid (Supplementary Tables S3b,c). This is a significant overrepresentation of genes with a peptidase domain in sterile relative to fertile hybrids (Z = 2.42; P = 0.016). Using *D. melanogaster* orthologs of the genes uniquely misregulated in sterile and fertile hybrids, we searched for those with a role in spermatogenesis or that have been identified as expressed in the sperm proteome. We found the same proportion of sperm/spermatogenesis genes (12%) among uniquely misregulated genes in sterile and fertile hybrids (Z = 0.022; P = 0.984) (Supplementary Table S4).

### Chromosomal distribution of misregulated genes in sterile hybrids

The chromosomal distribution of uniquely misregulated genes between fertile and sterile hybrids showed no bias (Table 2). A previous study identified four X-linked, one second and one third chromosome QTLs of both small and large effects on binary (none *vs* progeny produced) hybrid male sterility between *D. p. pseudoobscura* and *D. p. bogotana*^13^. We looked for genes uniquely misexpressed in the sterile hybrid that mapped within these QTLs. We identified one gene within a small effect X-chromosome QTL, four genes within the large effect second chromosome QTL and fourteen genes within the small effect third chromosome QTL (Table 3). We checked these genes for the presence of domains that might indicate involvement in reproduction-related biological processes or molecular functions (Table 3). Two processes which appear more than once are particularly interesting. Two genes (GA17404 and GA20821) have cell adhesion domains. Cell adhesion was proportionally enriched among misregulated genes in sterile hybrids (Fig. 2) and the *D. melanogaster* orthologs of GA17404 and GA20821 have high to very high levels of expression in the male accessory glands (http://flybase.org/). Accessory gland expressed proteins are known to trigger a variety of physiological responses in females after mating and the presence of cell adhesion domains is interesting in the context of possible male × female interactions. Three genes are peptidases and one is a peptidase inhibitor (Table 3). This class, as mentioned earlier, is overrepresented in sterile relative to fertile male hybrids. The fact that these four genes mapped to regions previously identified as significant in terms of hybrid male sterility further suggests a major role of previously unsuspected peptidases contributing to reproductive isolation between *D. p. pseudoobscura* and *D. p. bogotana*.

A previous study identified *Overdrive* (*Ovd*) as a major sterility gene for this species pair^14^. The *D. p. bogotana* X-linked allele in a hybrid genome background causes hybrid sterility, though the presence of other species-specific alleles at a number of other loci is also necessary^13^. This gene was not misexpressed in sterile or fertile hybrids in this study, though it was significantly differentially expressed between the two parental species.

### The contributions of cis and trans regulatory differences to divergence between species

**Figure 3:**
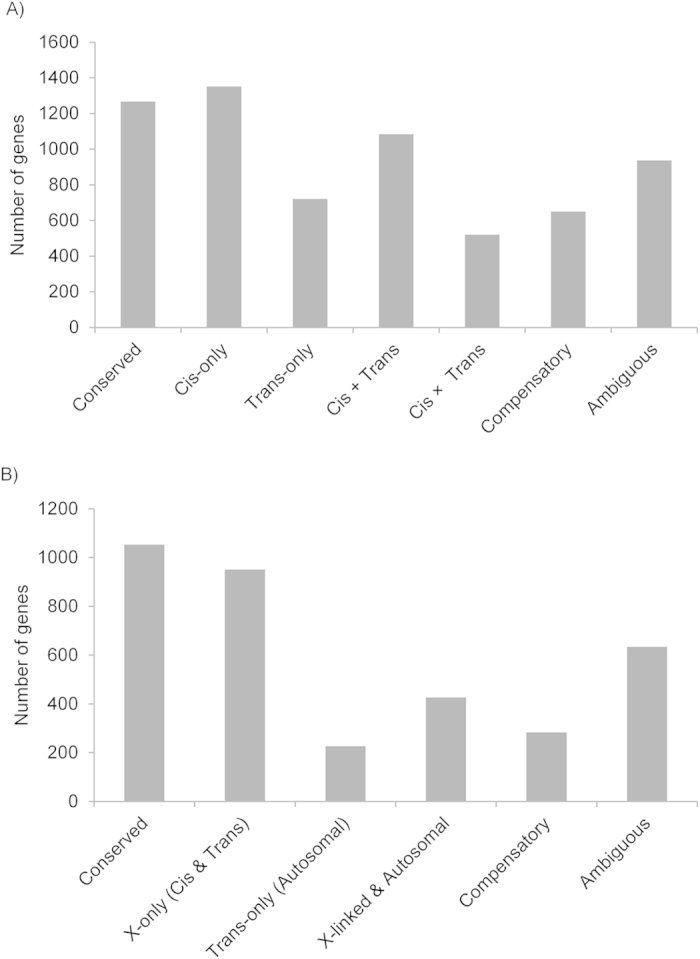
Identification of regulatory differences between species showing autosomal (A) and X-linked genes (B). The genes are further divided based on whether they showed evidence of significant divergence between species driven by loci that act in *cis* (Cis-only) or *trans* (Trans-only). Genes with significant evidence of both *cis*- and *trans*-regulatory differences were subdivided into “Cis + Trans” when differentially expressed genes between species favored expression of the same allele in the hybrids, “Cis × Trans” when differentially expressed genes favored expression of opposite alleles, and “Compensatory” for genes with no significant expression differences between species despite evidence for both *cis*- and *trans*-regulatory divergence. The term “Ambiguous” refers to genes with significant expression divergence between species but no significant evidence of *cis*- or *trans*-regulatory differences. For X-linked genes the classification is similar except that we could only identify effects as “X-only (Cis & Trans)”, meaning that the differences in expression between species is similar between hybrids despite the shared hybrid nature of their autosomes or “Trans-only (Autosomal)”, meaning that the differences in expression between species was not detected between hybrids due to the fact that one of the hybrids autosomes matches the species origin of the X-chromosome. “X-linked & Autosomal” refers to X-linked genes showing differential expression between species driven by a combination of both X-chromosome and autosomal regulatory elements (see Table 4).

Using SNP information, we assigned RNA sequence reads in hybrids to their parent of origin for 6,530 autosomal genes and 3,574 X-linked genes. In order to determine patterns of *cis* and *trans* regulatory divergence between species, we compared relative expression between parental species to relative allelic expression in the hybrids. For autosomal genes, we assayed expression in phenotypically normal (fertile) hybrids but, given that the X chromosome is hemizygous in males, we used information from both hybrids for X-linked genes. For autosomal genes, we found a significant deviation from equal proportions of *cis* (0.55) and *trans* effects (0.45) (binomial test, *P* < 0.001). *Cis*-only (21%) and *cis* + *trans* (17%) effects were overrepresented (Supplementary Table S2a) (Fig. 3A). For X-linked genes, we found a higher proportion of X-only (both *cis*- and X-linked *trans*-effects) followed by X-linked & autosomal effects. Though this could indicate a pattern similar to autosomal genes of divergence driven by *cis* (X-linked) effects, a definitive determination cannot be made by this method, as any diverged regulatory factors on the X cannot be assigned as *cis*-only or *trans*-only. (Supplementary Table S2b) (Fig. 3B).

**Figure 4:**
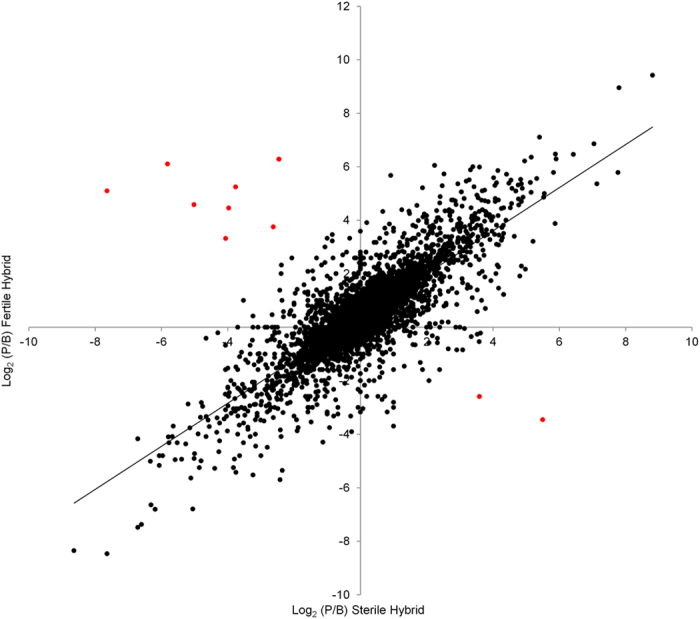
The ratio of parental allelic expression for each autosomal gene in fertile and sterile hybrids. Genes with the largest reversal in allelic expression between the two hybrids are indicated with a red circle.

In order to detect changes in allelic expression patterns in sterile relative to fertile hybrids, we compared the ratios of allelic expression across autosomal genes (Supplementary Table S2a). We found an overall positive correlation between hybrids (r = 0.798; P < 0.001) indicative of significant genome-wide conservation of allelic expression patterns, with a subset of genes having allelic ratios that significantly differed between hybrids (24% at q < 0.5% and 17% at q < 0.1%). Particularly striking are ten genes with the largest reversal in allelic expression between the two hybrids, with eight genes favoring the allele matching the X-chromosome genotype (Fig. 4). Seven of these eight have a mode of evolutionary divergence involving *trans* effects (i.e. compensatory and *cis* + *trans*).

**Figure 5:**
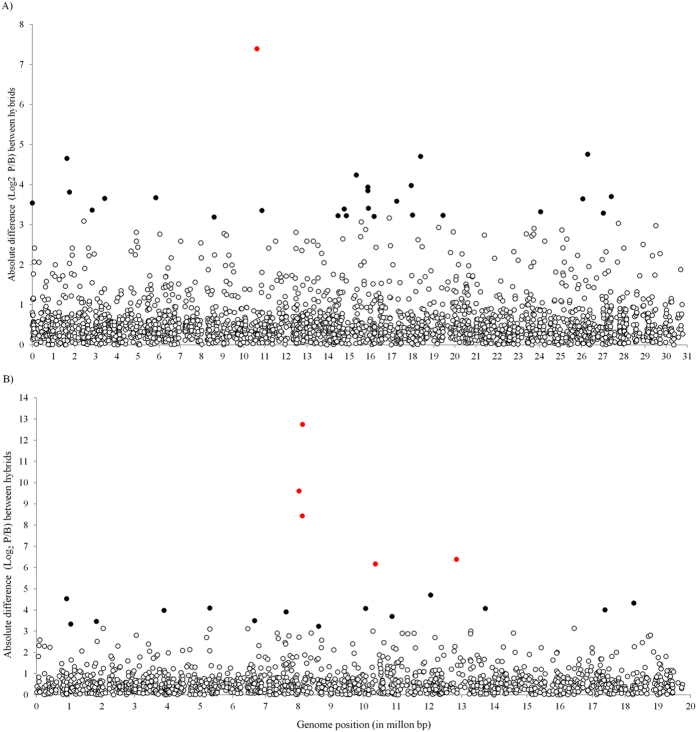
Absolute differences in allelic expression ratio between sterile and fertile hybrids. Scatter plot of second (**A**) and third (**B**) chromosome allelic ratio differences per chromosomal position. Filled black circles are the 1% of genes with the highest ratio differences. Genes with the largest reversal in allelic expression between the two hybrids are shown in red.

It is possible that large differences in allelic expression ratios between hybrids contribute to impaired fertility. We explored the intra- and inter-chromosomal distribution of sixty five genes in the top 1% of genes with the largest fold-change in allelic ratio between hybrids (at least a nine-fold change in allelic expression ratio) as well as their possible biological function. We detected no overrepresentation among these genes in terms of their GO biological process / molecular function, or the presence of particular polypeptide domains (Supplementary Table S3d). The proportion of these genes per million base pairs did not differ among the second, third and fourth chromosomes (0.87, 0.90 and 0.73 respectively) (Z = 1.56; P = 0.119 for third and fourth chromosomes comparisons). However, the distribution within the two fully assembled chromosomes (i.e. second and third) is not random (Fig. 5). Of the ten genes with the largest reversal in allelic expression between the two hybrids (Fig. 4), five genes are on the third chromosome, three of which are clustered within a 104 Kb chromosomal region (Fig. 5).

## Discussion

We have used RNA sequencing to map genome-wide differences in male reproductive tract expression between two closely related species of *Drosophila* and their hybrids. We chose the *D. p. pseudoobscura* and *D. p. bogotana* pair as they produce unidirectional hybrid male sterility^10^, thus allowing us to identify changes unique to the hybrid sterile condition.

Among genes that were misregulated only in the hybrids, four findings are of particular relevance: First, we found more uniquely misregulated genes in the sterile than the fertile condition, with a large proportion of genes displaying levels of expression outside the range of their parental species (i.e. transgressive expression). The deviation from additive effects among genes misregulated only in sterile hybrids is indicative of defective gene regulation, and this non-additive pattern is common among male-biased genes^15^,^16^,^17^. We found no evidence of an overrepresentation of down-regulation, as has been common for sterile hybrid males of other species of *Drosophila*^4^,^16^,^18^,^19^. The lack of overrepresented under-expressed genes cannot be explained by differences in divergence time between species pairs compared, as both species pairs within the *simulans* clade and the species pair we studied have diverged for approximately 0.25 Myr, while the *D. santomea* × *D. yakuba* pair have diverged for 0.4 Myr^11^,^20^,^21^. Instead, differences in the proportion of over- and under-expressed genes might be explained by the severity of sterility. Sterile male hybrids between species of *Drosophila* previously analyzed are at best unable to produce individualized sperm^22^,^23^, whereas sterile males between *D. p. pseudoobscura* and *D. p. bogotana* produce amotile individualized sperm cells^10^. Thus, it is possible to speculate that over expression is more frequent in sterile hybrids with a less severe sterility condition. In fact, Llopart^19^ found that the sterile *D. yakuba* × *D. santomea* hybrids that produced spermatids but no sperm have more overexpressed genes than the reciprocal hybrid with normal testis but no spermatids.

Second, we found no overrepresentation of sperm or spermatogenesis genes among those uniquely misregulated in the sterile hybrids, contrary to previous studies^2^,^4^. This is rather unexpected given that male reproductive genes in general and spermatogenesis genes in particular, have been shown to rapidly diverge at both coding and regulatory regions between species of *Drosophila*^2^,^24^,^25^,^26^,^27^,^28^,^29^. Once again, the lack of an overrepresentation of misregulated sperm/spermatogenesis genes might reflect the lack of spermatogenic developmental arrest in the *D. p. bogotana* × *D. p. pseudoobscura* sterile hybrid. This raises the question as to whether any other gene classes are uniquely misregulated in these sterile hybrids.

This brings us to our third point, our finding of significant overrepresentation of proteases among uniquely misexpressed genes in sterile hybrids. Misregulation of male reproductively expressed proteases has not been previously identified as a possible contributor to hybrid male sterility in *Drosophila*. The result is intriguing given recent studies on the reproductive role of male accessory gland proteases and protease inhibitors, as well as proteolytically active genes within the female reproductive tract of *D. melanogaster*^30^. Proteases are abundant throughout the male reproductive system, as well as within female reproductive tracts^31^,^32^ and while specific functions are not always known, several with fertility effects have been identified. The seminal fluid protease ‘seminase’ plays a key role in post-mating success, by causing ‘sex peptide’ to localize to sperm and by activating other proteins resulting in the gradual release of sperm from female storage, ovulation, and stimulation of egg laying^33^. Proteases also seem to be required for the acquisition of sperm motility in some species^34^,^35^. Male reproductive proteolytic proteins show evidence of rapid evolution; in a study comparing *D. mojavensis* and *D. melanogaster*, many genes showed species-specific proteolytic/protease inhibitor functions in the seminal fluid^36^. Similarly, the misexpressed proteases in our sterile hybrids may be specific to the *D. pseudoobscura* lineage - though one of our misexpressed genes, GA14907, is orthologous to a known sperm protein (S-Lap 5) in *D. melanogaster*, which is upregulated during meiotic and post-meiotic stages of sperm development^37^. The misexpression of proteolytic proteins in hybrids could contribute to sterility via tissue damage, especially in the case of overexpression. Of the 17 uniquely misexpressed proteases in the sterile hybrid, 13 were overexpressed and 3 of the 4 protease inhibitors were underexpressed. Misexpression of proteases can also contribute to sterility through disruption of important pathways in the male reproductive system, or improper post-mating interactions with female proteins^30^,^33^. We propose that male reproductive tract proteases and inhibitors might have evolved under different species-specific selective pressures between *D. p. pseudoobscura* and *D. p. bogotana*, in concert with their respective species-specific female tract proteolytic/inhibitor genes, resulting in hybrid regulatory incompatibilities that contribute to sterility. Interestingly, a group of four genes with peptidase/ peptidase inhibitor domains were within previously mapped hybrid male sterility loci^13^. These genes should serve as future candidates for gene-phenotype association studies.

Fourthly, we do not find an overrepresentation of uniquely misregulated X-linked genes in the sterile compared to the fertile hybrid, and the proportion of misregulated genes was slightly lower in the X chromosome than the autosomes. This indicates that gene-specific misregulatory effects have not accumulated on this chromosome per se. However the previously identified ‘large X effect’ explanation for hybrid male sterility in the *D. p. pseudoobscura* × *D. p. bogotana* cross^13^,^38^ could be a consequence of X-linked *trans*-regulatory elements contributing towards male sterility. *Ovd*, the only identified hybrid male sterility gene in crosses between *D. p. pseudoobscura* and *D. p. bogotana*, is an X-linked gene that contains a DNA binding (MADF) domain and has seven fixed non-synonymous differences between the two species^14^. These fixed protein differences together with the presence of a DNA binding domain and our finding of differential expression between parental species suggests that *Ovd* may contribute to sterility by acting as a transcription factor that causes the misregulation of downstream genes. A potentially disproportional effect of X-linked *trans* regulatory gene divergence is also hinted at by the fact that eight out of ten autosomal genes with the largest reversal in allelic expression between hybrids favored the allele matching the X-chromosome genotype.

Our results also provide a first glance at genome-wide regulatory divergence between these two species. We found a preponderance of *cis* rather than *trans* divergence. This is in line with several studies showing a high proportion of interspecies *cis* regulatory divergence^39^,^40^,^41^. The dominant pattern of *cis*-only regulatory divergence between the two *D. pseudoobscura* species is particularly interesting, as *cis*-only changes affect mostly the expression of genes at terminal steps of regulatory networks or genes not involved in large interactive networks^42^. A study comparing *trans* and *cis* regulatory changes within *D. pseudoobscura* using females had found more *trans* than *cis* mutations within species^43^. This is expected as there are more genome-wide *trans* than *cis* mutational targets, but because *trans* acting mutations can affect the expression of many genes, selection would tend to eliminate the accumulation of such mutations over time. This form of purifying selection eliminating *trans* mutations would be detectable among closely-related species, like the species pair in our study. We also detect a higher genome-wide proportion of *cis* + *trans* (16.6%) than *cis* x *trans* antagonistic (8%) divergence. This could be explained by directional selection between species favoring alleles with additive *cis*-only and agonistic *cis* + *trans* effects^17^,^18^,^44^. The paucity of uniquely misregulated transgressively expressed genes in sterile hybrids also aligns with the paucity of *cis* x *trans* antagonistic regulatory divergence (8%). Finally, despite a similar pattern of allelic expression between sterile and fertile hybrids, some individual genes show large differences in expression. One particular region of the third chromosome has three closely mapped genes with very high fold changes in allelic ratios between hybrids. One gene has a spindle associated domain, and another a proteasome complex domain. These domains have been associated with sperm differentiation function^45^,^46^ and although it is premature to speculate, the chromosomal proximity between genes, the presence of potential sperm differentiation genes among them and the large change in allelic expression ratio between hybrids highlights these genes as interesting targets for future gene function analysis in these species.

## Methods

### Species selection and stock maintenance

Stocks used for this study were obtained from the UCSD *Drosophila* Stock Centre (https://stockcenter.ucsd.edu/): *D. p. pseudoobscura* (14011–0121.139) and *D. p. bogotana* (14011-0121.175). Flies were maintained on cornmeal–molasses–yeast–agar (CMYA) medium at constant temperature (24 °C) on a 12 hour light–dark cycle. Virgin females were collected post-eclosion and flies were mass crossed in bottles containing CMYA medium. Reciprocal crosses were used to create hybrids.

### Sample Preparation and Sequencing

Total RNA was extracted from reproductive tracts (testes, accessory glands, and ejaculatory bulb) using the Qiagen RNeasy Plus kit. Two biological replicates were obtained from parental species and hybrids, each sample consisting of 30 to 40 male reproductive tracts. RNA samples were tested for quality using an Agilent Bioanalyzer and quantified using a nanodrop (Thermo Scientific). RNA samples were sent to the McGill University and Génome Québec Innovation Centre for library preparation and sequencing (http://gqinnovationcenter.com/). Briefly, cDNA libraries were prepared using Illumina’s TruSeq Stranded mRNA sample preparation kit from 250 ng of total RNA, followed by 100 bp paired-end sequencing. All eight samples were run multiplexed on a single lane of an Illumina HiSeq2000 machine.

### Mapping and Differential Expression Analysis

After sequencing, reads were adaptor-trimmed using the Trimmomatic program^47^. Any paired reads in which either the forward or reverse read was shorter than 50 bp after trimming were discarded. The Tuxedo tool suite was used for gene expression analysis, following steps previously outlined but excluding the final CummeRbund step^48^. Tools were run *via* the Galaxy platform (https://usegalaxy.org/). Because there is no sequenced genome for *D. p. bogotana*, reads from all samples were aligned to version 3.1 of the *D. p. pseudoobscura* genome from Flybase (http://flybase.org/) using the short-read gap-junction aligner TopHat^48^. A maximum of 8 mismatches to the genome were allowed during mapping (Supplementary information).

Aligned reads were assigned to a gene of origin by Cufflinks, using the *D. p. pseudoobscura* V3.1 annotation as a guide for transcript assembly. The annotation was filtered to include only genes annotated as ‘Flybase’. Pair-wise differential expression testing between each parental species and reciprocal hybrid was conducted using Cuffdiff. Cuffdiff reports expression levels as fragments per kilobase of exon per million mapped reads (FPKM), which controls for gene length and per-sample sequencing depth. Cuffdiff conducts significance testing using a beta negative binomial distribution, At least one sample with ten mapped reads was required for significance testing, with FDR correction set at 0.05. We used the FDR corrected pairwise comparisons from CuffDiff to identify genes differentially expressed between the two parental species and hybrids. Genes differentially expressed in the hybrids were classified as additive or transgressive (i.e. genes with expression higher or lower than the parental species).

Genes misexpressed in hybrids were assigned functional classifications using Gene Ontology biological processes, molecular function and identification of polypeptide domains (Interpro) within Flybase (http://flybase.org).

### Allele-Specific Gene Expression

The relative contribution of *cis* and *trans* interspecies divergence on gene expression was inferred using species-specific SNPs and relative allelic expression in the F1 fertile hybrid^18^,^39^. We utilized expression data from the fertile F1 male hybrids to avoid condition-specific (sterility) effects in assaying overall regulatory divergence.

SNPs identified between the two parental species using Naïve variant caller and Variant annotator^49^ were considered fixed if each parental species was represented by a different single allele, with at least 3 reads supporting each parent. RNA-seq reads in the hybrid samples were assigned to a parent of origin based on the identity of the allele at fixed SNP positions in each parent. Counts of all reads containing fixed SNPs mapping to a given gene ID were summed, and any gene IDs with at least 20 supporting reads in the two parental samples combined were retained. Counts were normalized to reflect differences in sequencing depth between samples. Any samples with zero reads mapping were adjusted to one in order to allow statistical testing. Relative contributions of mapped reads were calculated and significant differences in expression between parents (binomial exact test), between alleles in the hybrid (binomial exact test) and between the ratio of parental read counts to counts of each parental allele in the hybrid (Fisher’s exact test), done as in McManus *et al.*^18^. FDR corrected q-values were used for all three tests (significance q < 0.5%).

For X-linked genes we examined regulatory divergence by comparing SNP counts in the fertile and sterile hybrids. If regulatory factors on the X-chromosome (either *cis* or *trans*) are responsible for parental species expression divergence, then each hybrid will experience maternal-species levels of gene expression, and the ratio of parental expression will equal that of the ratio between the two hybrids. If autosomally derived *trans* factors alone are responsible for misexpression, then the sterile and fertile hybrid should express the X-linked gene in equal amounts, and the parental and hybrid ratios will differ (Table 4).

Finally, we compared changes in ratios of autosomal allelic expression in the hybrids using both correlations as well as Fisher’s exact tests in search of genome-wide patterns of differential allelic expression. We also examined the chromosomal distribution as well as potential function of the 1% of genes with the highest fold-change in allelic expression ratio between hybrids.

## Additional Information

**How to cite this article**: Gomes, S. and Civetta, A. Hybrid male sterility and genome-wide misexpression of male reproductive proteases. *Sci. Rep.* **5**, 11976; doi: 10.1038/srep11976 (2015).

## Supplementary Material

Supplementary Information

Supplementary Table S1

Supplementary Table S2

Supplementary Table S3

Supplementary Table S4

## Figures and Tables

**Table 1:** Sequencing depth per condition, before and after adaptor trimming.

Sample	Total	Trimmed	Mapped
*Dpse*	104,175,212	103,732,186	75,529,748
*Dbog*	104,982,638	104,587,190	77,445,307
*Dpse*×*Dbog*	110,052,924	109,497,908	80,014,206
*Dbog*×*Dpse*	104,970,274	104,571,170	77,329,355

The number of reads per condition is reported, with each forward and reverse read counted individually.

**Table 2:** Chromosomal distribution of uniquely misexpressed genes in sterile and fertile hybrids.

Chr	Genes	Sterile	Fertile	P (Z-test)
X	4,252	41 (0.0096)	11 (0.0026)	0.226
2	3,099	38 (0.0123)	20 (0.0065)	0.184
3	2,319	30 (0.0129)	12 (0.0052)	0.897
4	2,640	36 (0.0136)	9 (0.0034)	0.180
U	1,618	19 (0.0117)	12 (0.0074)	0.142
Total	13,928	164	64	

U = unmapped genes. The proportion of misregulated genes per total number of genes in each chromosome is shown in parentheses. *P* values are for per chromosome pair-wise comparisons between hybrids.

**Table 3:** Genes uniquely misexpressed in the sterile hybrid within previously mapped hybrid male sterility loci (Phadnis *et al.* 2011).

Ch	Gene ID	Gene	Position	*D. mel* Ortholog	Misreg	Bio Proc/ Mol func
XL	014414	GA13658	1955371	CG15343	Under	Oxidation reduction
2	003560	GA17404	22581928	*mfas*	Over	**Cell adhesion**
2	001942	GA20583	28920232	*DNaseII*	Over	Metabolic process
2	002011	–	30022325	–	Under	–
2	004037	GA19748	30740414	*Esyt2*	Over	Signal transduction, Membrane trafficking
3	004295	–	3262010	–	Over	–
3	005930	–	3403695	–	Over	–
3	006019	GA11668	4428405	*Arc1*	Over	Nucleic acid binding
3	006038	GA15650	4762673	CG30101	Under	?
3	004432	GA15652;GA18461	4822601	*NT5E-2*;*veil*	Under	Hydrolase activity; Hydrolase activity
3	004450	GA15722	5031658	CG30197	Over	**Peptidase inhibitor**
3	004080	GA20821;GA24606	523789	*scb*	Over	**Cell adhesion**; ?
3	005712	–	698906	–	Over	–
3	006249	GA21772	7424544	CG9416	Over	**Peptidase**
3	004638	GA24794	7431435	CG10062	Over	**Peptidase**
3	006251	GA24796	7451117	CG10073/81	Under	**Peptidase**
3	004044	–	78661	–	Additive	–
3	006299	–	7978456	–	Under	–
3	004848	GA20811	9673208	*ana*	Under	?

Dashes identify unknown genes. Question marks are genes with unknown function. Bolded are functions/processes linked to more than one gene ID.

**Table 4:** Types of regulatory divergence indicated by patterns of allelic expression of (a) autosomal genes, and (b) X-linked genes.

Divergence	P *vs* B	H_P_ *vs* H_B_	P/B *vs* H_P_/H_B_
a)			
Conserved	NS	NS	NS
*Cis* only	S	S	NS
*Trans* only	S	NS	S
Compensatory	NS	S	S
*Cis* & *Trans*	S	S	S
*Cis* + *Trans*	Same		
*Cis* × *Trans*	Opposite		
b)			
Conserved	NS	NS	NS
X-only (*Cis* & *Trans*)	S	S	NS
*Trans*-only (Autosomal)	S	NS	S
X-linked & Autosomal	S	S	S
Compensatory	NS	S	S

P = *D. p. pseudoobscura*, B = *D. p. bogotana*, H = F1 hybrids, with first initial of maternal species in subscript. NS = Non-significant differences in number of sequence reads between samples. S = Significant differences in number of sequence reads between samples. Same = the same allele (P or B) is more abundant in both parental and hybrid comparisons. Opposite = the more abundant allele in parental species comparisons becomes the less abundant allele in the hybrid.
