# Supplementary material for: Hybrid male sterility and genome-wide misexpression of male reproductive proteases
Source: Sci Rep. 2015 Jul 6;5:11976. doi: 10.1038/srep11976 (PMC4491705; doi:10.1038/srep11976)
Supplement: Supplementary Information [file srep11976-s1.pdf]

Supplementary Information

Hybrid male sterility and genome-wide misexpression of male reproductive proteases

Suzanne Gomes and Alberto Civetta*

Department of Biology, University of Winnipeg

515 Portage Ave, Winnipeg, MB, Canada

R3B 2E9

*Corresponding Author: Alberto Civetta, Department of Biology, University of Winnipeg

515 Portage Ave. Winnipeg, Manitoba, Canada. R3B 2E9

Tel: (204) 786-9436; E-mail: a.civetta@uwinnipeg.ca

Supplementary Methods: Settings used for each program in the Tuxedo Suite, all run via the Galaxy interface.

TopHat Settings

Input Parameter	Value
Is this library mate-paired?	Paired-end
Mean Inner Distance between Mate Pairs	100
TopHat settings to use	full
Library Type	FR First Strand
Std. Dev for Distance between Mate Pairs	825
Anchor length (at least 3)	8
Maximum number of mismatches that can appear in the anchor region of spliced alignment	0
The minimum intron length	50
The maximum intron length	175000
Allow indel search	Yes
Max insertion length.	3
Max deletion length.	3
Maximum number of alignments to be allowed	20
Minimum intron length that may be found during split-segment (default) search	50
Maximum intron length that may be found during split-segment (default) search	175000
Number of mismatches allowed in the initial read mapping	2
Number of mismatches allowed in each segment alignment for reads mapped independently	2
Minimum length of read segments	25
Use Own Junctions	No
Use Closure Search	No
Use Coverage Search	Yes
Minimum intron length that may be found during coverage search	50
Maximum intron length that may be found during coverage search	20000
Use Microexon Search	No

Cufflinks Settings

Input Parameter	Value
Max Intron Length	175000
Min Isoform Fraction	0.1
Pre MRNA Fraction	0.15
Perform quartile normalization	Yes
Use Reference Annotation	Use reference annotation guide
Perform Bias Correction	No
Use multi-read correct	No
Use effective length correction	Yes

Cuffmerge Settings

Input Parameter	Value
Use Reference Annotation	Yes
Use Sequence Data	No

Cuffdiff Settings

Input Parameter	Value
Library normalization method	geometric
Dispersion estimation method	pooled
False Discovery Rate	0.05
Min Alignment Count	10
Use multi-read correct	No
Perform Bias Correction	No
Include Read Group Datasets	Yes
Set Additional Parameters? (not recommended for paired-end reads)	No
